# Optimized Anti–Prostate‐Specific Membrane Antigen Single‐Chain Variable Fragment–Loaded Nanobubbles as a Novel Targeted Ultrasound Contrast Agent for the Diagnosis of Prostate Cancer

**DOI:** 10.1002/jum.15155

**Published:** 2019-11-08

**Authors:** Yu Ding, Qifeng Cao, Subo Qian, Xiaolong Chen, Yuhong Xu, Jian Chen, Haibo Shen

**Affiliations:** ^1^ Department of Urology, Xinhua Hospital, School of Medicine Shanghai Jiao Tong University Shanghai China; ^2^ Zhejiang California International NanoSystems Institute Zhejiang University Hangzhou China; ^3^ School of Pharmacy Shanghai Jiao Tong University Shanghai China

**Keywords:** molecular imaging, nanobubbles, prostate cancer, prostate‐specific membrane antigen, ultrasound contrast agents

## Abstract

**Objectives:**

To prepare optimized prostate‐specific membrane antigen (PSMA) single‐chain variable fragment (scFv)–loaded nanobubbles (NBs) as a novel targeted ultrasound (US) contrast agent for diagnosis and treatment of prostate cancer (PCa).

**Methods:**

Prostate‐specific membrane antigen scFv‐loaded NBs were prepared by membrane hydration and biotin‐streptavidin conjugation. Flow cytometry was used to observe the binding rate of the targeted NBs to PSMA‐expressing cells. Contrast‐enhanced US was used to monitor targeted and nontargeted NBs administered to nude mice with 22RV1, LNCaP, and PC‐3 xenograft tumors. The specific binding ability of the targeted NBs was further examined by fluorescence imaging of tumor cryosections.

**Results:**

Uniformly sized targeted NBs were successfully prepared (mean ± SD, 485.3 ± 28.4 nm). The NBs showed good stability and bound specifically to LNCaP and 22RV1 cells with high PSMA expression in vitro but did not bind to PC‐3 cells without PSMA expression. The targeted NBs presented good US enhancement, and the results of the in vivo xenograft tumor nude mouse model showed that the peak contrast intensity in LNCaP and 22RV1 cells was significantly higher for the targeted NBs than the nontargeted NBs (*P* < .05), whereas there was no significant difference in PC‐3 cells. Immunofluorescence results obtained from tumor sections confirmed that the targeted NBs were capable of targeting PSMA‐expressing tumor cells.

**Conclusions:**

These novel PSMA scFv‐loaded NBs have proven to be an excellent US contrast agent for imaging PSMA‐expressing PCa and have the potential to not only enable efficient and safe molecular imaging but also to serve as a delivery system for targeted PCa therapies.

**Abbreviations**
biotinylated DSPE‐PEG‐2000: 1,2‐distearoyl‐sn‐glycero‐3‐phosphoethanolamine‐*N*‐[biotinylated (polyethylene glycol)‐2000]
CEUS: contrast‐enhanced ultrasound
DPPC: 1,2‐dipalmitoyl‐sn‐glycero‐3‐phosphocholine
FCM: flow cytometry
MB: microbubble
NB: nanobubble
PBS: phosphate‐buffered saline
PCa: prostate cancer
PEG: polyethylene glycol
PSMA: prostate‐specific membrane antigen
scFv: single‐chain variable fragment
UCA: ultrasound contrast agent
US: ultrasound

Prostate cancer (PCa) has become the most common cancer in men in recent years. In 2016, there were 1.4 million cases of PCa and 381,000 deaths worldwide.^1^ Early diagnosis is important for the treatment and prognosis of patients with PCa.^2^ Transrectal ultrasound (US)‐guided biopsy is the reference standard for diagnosing PCa, and the application of an ultrasound contrast agent (UCA) improves the diagnostic sensitivity and accuracy of this method.^3^ Microbubbles (MBs), a commonly used UCA, are bubbles larger than 1 μm in diameter and have the advantages of low cost, easy preparation, and excellent contrast.^4^ However, because of size limitations, MB‐based imaging is limited to the vasculature, and the MBs usually pool in the blood, rather than circulating as desired.^5^ Thus, nanobubbles (NBs) have recently become a hot topic in cancer contrast imaging research. The neovasculature of a tumor is imperfect and has a maximum pore size in the wall of approximately 380 to 780 nm.^6^ Nanobubbles can pass through small pores in the vascular endothelium that MBs cannot pass through and reach the tumor parenchyma. In addition, NBs have the advantages of stronger stability, better blood flow, and a longer in vivo imaging time for tumor enhancement.^7^, ^8^, ^9^

In recent years, the development of US molecular imaging has further enhanced the efficacy of contrast‐enhanced ultrasound (CEUS) tumor imaging. Targeted NBs are prepared by loading a specific ligand onto the surface of the NBs, which can then circulate through the blood and pass through the vascular endothelium to reach the target tissue; here, the NBs can aggregate and persist for a long time. Studies have shown that targeted NBs can increase the peak time and intensity values of tumor imaging in vitro compared to nontargeted NBs.^10^, ^11^, ^12^ Prostate‐specific membrane antigen (PSMA), which was discovered in 1987, is a unique intact transmembrane type 2 glycoprotein in prostate epithelial cells.^13^ Its expression is positively correlated with the mortality rate of PCa^14^ and is considered the most important protein target in diagnostic‐specific immunolocalized imaging and immunoguided therapy.^15^, ^16^

Lipid shells are the most classic component of diagnostic contrast agents and have the advantages of excellent elasticity, easy modification, and biocompatibility.^17^ Meanwhile, adding polyethylene glycol (PEG) can delay NB clearance by the reticuloendothelial system.^4^ At present, there are few studies on PSMA antibody‐targeted NBs and its US‐targeting development. Differential centrifugation and static stratification are usually used to construct NBs, which are not conducive to the yield and stability of NBs, and their targeting is still lacking. On the basis of these advances, in this study, we prepared stable octafluoropropane gas core and phospholipid‐PEG shell NBs of a uniform size and then loaded them with a PSMA single‐chain variable fragment (scFv) using biotin‐streptavidin conjugation to produce targeted NBs for the imaging of tumors overexpressing PSMA.

## Materials and Methods

### *Materials*

1,2‐Dipalmitoyl‐sn‐glycero‐3‐phosphocholine (DPPC; molecular weight, 734 g/mol) and 1,2‐distearoyl‐sn‐glycero‐3‐phosphoethanolamine‐*N*‐[biotinylated (polyethylene glycol)‐2000] (biotinylated DSPE‐PEG‐2000; molecular weight, 2967 g/mol) were purchased in powder form from NanoSoft Polymers (Winston‐Salem, USA) and used without further purification. Octafluoropropane gas was purchased from Keyuan Gas (Chengdu, China); biotin‐PEG‐maleimide was purchased from Nanocs (Boston, MA); and streptavidin was purchased from Thermo Scientific (Shanghai, China). The anti‐PSMA scFv was donated by the Zhejiang California International NanoSystems Institute, Zhejiang University, the laboratory of the School of Pharmacy of Shanghai Jiaotong University. The PSMA scFv was constructed according to a study reported by Friedrich et al^18^ and contained a histidine tag.

### *Preparation of Biotinylated NBs*

Biotinylated NBs were prepared by a thin‐film hydration method. First, a total of 33 mg of DPPC and biotin‐DSPE‐PEG‐2000 were weighed at a certain ratio (9:1, molar ratio), mixed into a 25‐mL round‐bottom glass bottle, and dissolved in 2 mL of chloroform. A uniformly distributed dry thin‐film mixture was then formed at the bottom of the glass bottle by rotary evaporation (55°C, 10 minutes, 130 rpm) with a rotary evaporator (Shensheng Biotechnology, Shanghai, China). Then, Tris, propylene glycol, and glycerol were mixed at a certain ratio to prepare a hydration solution of pH 7.2; 6 mL of the mixture was then added to the above glass bottle and subjected to US sonication in a bath at 45°C for 30 minutes to prepare a liposomal film suspension. The suspension was then placed in vials sealed with a rubber cap, which were pumped with octafluoropropane gas through a slender needle after vacuum pumping. Last, the samples were oscillated for 60 second using an ST series capsule mechanical oscillator (Antai Biomedical Materials, Beijing, China), and the formed NBs were immediately stored at 4°C. The NB stock solution was then removed and diluted 10 times with sterile ultrapure water. The diameter and zeta potential of the NBs were determined with a Zetasizer Nano ZS90 analyzer (Malvern Panalytical, Malvern, England); the concentration of the NBs was determined with a NanoSight NS300 nanoparticle‐tracking analyzer (Malvern Panalytical).

### *Preparation of Biotinylated Antibodies*

After 150 μg of the anti‐PSMA scFv was mixed with 100 μL of Tris (2‐carboxyethyl) phosphine solution, the mixture was purged of oxygen with nitrogen and allowed to react at room temperature for 45 minutes to reduce the thiol group in the antibody. Then, 50 μg of biotin‐PEG‐maleimide 600 was added and placed on a shaker for 16 hours (4°C, 350 rpm). Subsequently, the reaction product was transferred to an ultrafiltration tube (10 kDa) and filtered 3 times with phosphate‐buffered saline (PBS). The optical density of the obtained solution was determined by measuring the absorbance at 280 nm to calculate the antibody concentration. The biotin concentration in the sample was calculated with a biotin quantification kit (Thermo Scientific). Finally, the biotin‐to‐antibody coupling ratio was calculated.

### *Preparation of Targeted NBs*

The prepared biotinylated NB stock solution was diluted 10 times with sterile ultrapure water and then mixed in a ratio of 10^8^ NBs to 300 μg of streptavidin. The mixture was allowed to react at 4°C for 1 hour and was then washed (300 rpm, 2 minutes) 3 times to remove excess streptavidin. The biotinylated PSMA antibody prepared above was then added and mixed in a ratio of 10^8^ NBs to 15 μg of the biotinylated PSMA antibody. The mixture was stored at 4°C for 1 hour and then washed (300 rpm, 2 minutes) 3 times to remove the excess biotinylated PSMA antibody, yielding targeted NBs. The diameter and zeta potential of the targeted NBs were determined with the Zetasizer Nano ZS90 analyzer; the concentration of the targeted NBs was determined with the NanoSight NS300 nanoparticle analyzer.

### *In Vitro Characteristics of Targeted NBs*

The prepared targeted NB solution was dispensed into a 1‐mL glass vial at room temperature, and the NB diameter and concentration were measured at 10, 30, 60, 90, and 120 minutes. Prostate‐specific membrane antigen–targeted NBs in a 5‐mL Eppendorf tube were imaged in vitro by US using a color Doppler US system (LOGIQ E9; GE Healthcare, Chicago, IL) in the CEUS mode with an ML6‐15 transducer and a mechanical index of 0.07. After a sufficient amount of US couplant was applied to the surface of the Eppendorf tube, 4 mL of PBS was added to the tube for observation; then, 1 mL of both the notargeted and targeted NB suspensions were added. Ultrasound imaging was performed at room temperature.

### *Construction of In Vitro Cell Culture and In Vivo Tumor Xenograft Models*

22RV1 cells (androgen‐independent human PCa cells) and PC‐3 cells (androgen‐independent human PCa cells) were obtained from the Department of Urology of Xinhua Hospital. LNCaP cells (androgen‐dependent human PCa cells) were obtained from the Shanghai Institute of the Chinese Academy of Sciences Cell Bank (Shanghai, China). The cells were cultured at 37°C in 5% carbon dioxide. PC‐3 cells were cultured in Dulbecco's modified Eagle's medium–F12 containing 10% heat‐inactivated fetal bovine serum; 22RV1 and LNCaP cells were cultured in RPMI 1640 culture medium containing 10% heat‐inactivated fetal bovine serum. Cells of each type were passaged with 0.25% trypsin every 3 to 5 days, all in the logarithmic growth phase. BALB/c nude mice (male, 3–6 weeks old, 20 g) were provided by the Laboratory Animal Center of Xinhua Hospital. After 150 μL of a suspension of tumor cells in the logarithmic growth phase (2 × 10^7^/mL) and 150 μl of Matrigel (BD Biosciences, San Jose, CA) were mixed, the mixture was subcutaneously injected into the gluteal hip of nude mice to form a tumor xenograft model. Animals were housed in a clean environment and tested according to international guidelines. The animal experiments involved in this study were approved by the Animal Ethics Committee of Xinhua Hospital, affiliated with Shanghai Jiao Tong University School of Medicine.

### *Immunofluorescence and Specific Targeting of Targeted NBs In Vitro*

22RV1, LNCaP, and PC‐3 cells in the logarithmic growth phase were seeded in the wells of a 24‐well plate covered with coverslips at 1 × 10^4^ cells per well; then, the test solution was added. The plates were then incubated at 37°C in 5% carbon dioxide overnight. The solution was removed the next day, and the cells were washed 3 times with PBS. Then, the cells were fixed in 4% paraformaldehyde for 20 minutes, washed 3 times with PBS, and blocked with 1% bovine serum albumin at room temperature. A 1:100 dilution of the Alexa Fluor 488–labeled anti‐PSMA antibody (Abcam, Cambridge, England) was added, and the cells were incubated for 1 hour at 37°C in the dark. Finally, the cells were washed with PBS 3 times, stained with 4′,6‐diamidino‐2‐phenylindole, washed with PBS 3 times again, and then covered to be observed by fluorescence microscopy.

Flow cytometry (FCM) was performed to determine the rate of conjugation between the targeted NBs and the cells. 22RV1, LNCaP, and PC‐3 cells cultured as described above were collected into a sterile test tube. The cell concentration was measured with a hemocytometer, and 2 × 10^5^ cells of each type were placed into 3 sterile flow tubes corresponding to 3 groups: PBS (500 μL) was added to tube 1 as a nontargeted control; nontargeted NBs (500 μL) were added to tube 2; and targeted NBs (500 μL) were added to tube 3. Because the cells would sink because of the effect of gravity, the gravity of NBs containing gas was lighter, and they would rise. Therefore, 3 groups of aseptic flow tubes were placed on a shaking table and reacted at room temperature for 45 minutes. Then, after the cells were centrifuged at 20*g* for 3 minutes 3 times, the DyLight 488–labeled anti‐6X histidine‐tagged antibody (ab117512; Abcam) was added, followed by incubation for 15 minutes in the dark. The supernatant was discarded after the samples were centrifuged 3 times. Finally, the cells were resuspended in 1× PBS before FCM.

### *Ultrasound Imaging of Tumor‐Bearing Mice*

When the volume of the subcutaneous 22RV1, LNCaP, and PC‐3 cell‐derived tumors in the nude mice reached approximately 1 cm^3^, each nude mouse was anesthetized by an intraperitoneal injection of 100 μL of 1% sodium pentobarbital and fixed to a plate. A sufficient amount of US‐transmissive gel was applied to the top of the tumor to fully observe the xenografted tumor by US. The size of the tumor was measured by CEUS using a dynamic US system (LOGIQ E9) with an ML6‐15 transducer and a mechanical index of 0.07. Throughout the experiment, all parameters remained unchanged. Nontargeted NBs and targeted NBs were diluted to 1 to 3 × 10^8^ NBs/mL with saline. Real‐time acquisition began before the NB injection. In each nude mouse, 200 μL of nontargeted NBs was injected through the retro‐orbital sinus, and 200 μL of targeted NBs was injected after an interval of more than half an hour. During this time, the flash technology of the US instrument was used to destroy residual nontargeted NBs in the body. The arrival time, time to peak, and duration of contrast enhancement were observed. Peak‐intensity grayscale images were analyzed with the the WCIF ImageJ software program (National Institutes of Health, Bethesda, MD).

### *Fluorescence Imaging of Tumor Cryosections*

22RV1, LNCaP, and PC‐3 tumor‐bearing mice were randomly divided into 2 groups: nontargeted and targeted NB groups. First, 3 mL of 1,1′‐dioctadecyl‐3,3,3′,3′‐tetramethylindocarbocyanine perchlorate (1.2 mg/mL) was added to 1 mL of a suspension of nontargeted or targeted NBs for 5 minutes to dye the NBs. Then, the mixture was centrifuged at 20*g* for 3 minutes, and the supernatant was collected. Each mouse in both groups was then injected with 200 μL (1–3 × 10^8^ NBs/mL) of nontargeted or targeted NBs through the retro‐orbital sinus. Five minutes later, the tumors were extracted for cryosectioning and examination.

## Results

### *Coupling of Biotin and the Antibody*

The absorbance of the biotinylated antibody at 280 nm was 1.23, as determined with an ultraviolet spectrophotometer (Infinite M200 Pro; Tecan, Shanghai, China). The antibody concentration was calculated as 1 mg/mL, and the molar concentration was 3.57 × 10^5^ mol/L.

The molar concentration of biotin in the antibody solution was measured with a Pierce biotin quantitation kit (Thermo Scientific) to be 5.00 × 10^5^ mol/L. The biotin‐to‐antibody coupling ratio was 1.4:1.

### *Characterization of Biotinylated NBs and Targeted NBs*

**Figure 1:**
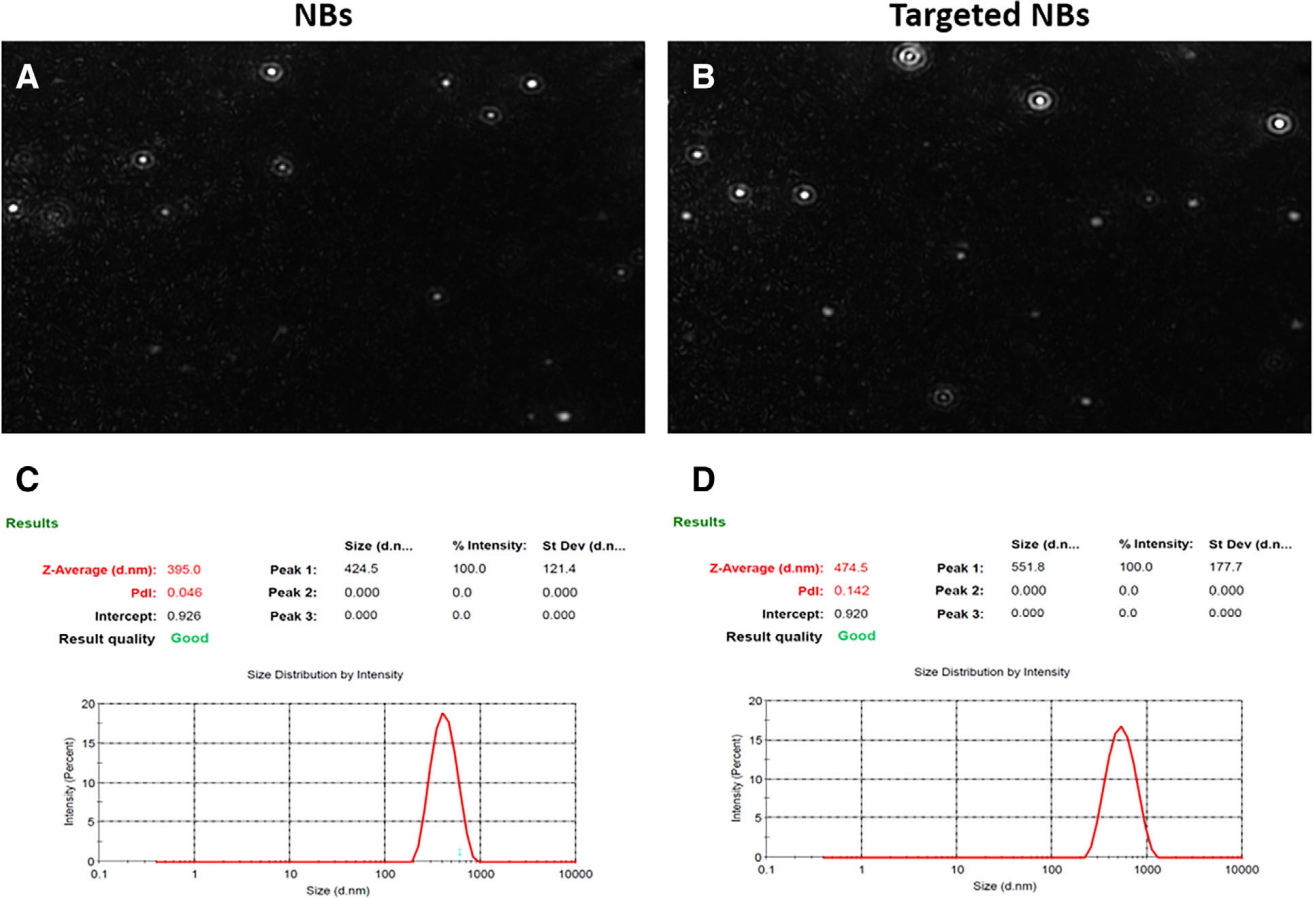
**A** and **B**, Observation of NBs (**A**) and targeted NBs (**B**) under the NS300 nanoparticle‐tracking analyzer. **C** and **D**, Characteristics of the size distribution of NBs (**C**) and targeted NBs (**D**). PdI indicates polydispersity index a measure of polymer molecular weight distribution. The smaller the value, the more uniform the molecular weight distribution.

A white suspension was obtained after the preparation of nontargeted biotinylated NBs. The prepared nontargeted biotinylated NB stock solution was diluted 10 times with sterile ultrapure water and examined with the NS300 system. Nanobubbles of a uniform size and round shape were observed; they showed no aggregation. The particle size distribution (Figure 1) and zeta potential of the biotinylated NBs were then evaluated. The mean diameter of the NBs ± SD was 414.6 ± 30.5 nm; the polydispersity index was 0.046 ± 0.012; and the zeta potential was –20.31 ± 5.7 mV. The targeted biotinylated NBs were not significantly different from the nontargeted biotinylated NBs in terms of morphologic characteristics, as observed with the the NS300 system. However, the mean diameter of the targeted NBs was 485.3 ± 28.4 nm, which was larger than the value recorded for the nontargeted biotinylated NBs (*P* < .05). The polydispersity index was 0.136 ± 0.28, and the zeta potential was –16.58 ± 3.9 mV (n = 3). The concentrations of the nontargeted and targeted NBs measured with the NS300 system were 3.17 × 10^9^ ± 6.67 × 10^8^ and 2.12 × 10^9^ ± 5.31 × 10^8^ NBs/mL, respectively.

### *In Vitro Stability and US Imaging*

We tested the stability of the targeted NBs at room temperature with a total of 10 samples. The size of the targeted NBs was slightly increased from 485.3 ± 28.4 to 565.5 ± 19.9 nm after 60 minutes (*P* > .05). However, the size of the targeted NBs gradually increased (664.7 ± 30.9 nm) after 90 minutes to be significantly larger than newly prepared targeted NBs (*P* < .05). Nevertheless, all measured diameters did not exceed approximately 800 nm. After 120 minutes, the targeted NBs formed larger bubbles (986.3 ± 116.8 nm), some exceeding the nanometer size range. Similarly, after 60 minutes, the concentration of targeted NBs (1.68 × 10^9^ ± 3.69 × 10^8^ NBs/mL) was not significantly different from the initial concentration (2.12 × 10^9^ ± 5.31 × 10^8^ NBs/mL; *P* > .05). However, after 90 minutes, the concentration (7.6 × 10^8^ ± 4.92 × 10^7^ NBs/mL) was significantly lower than the initial concentration (*P* < .05). Thus, we tested the binding capability and US contrast‐enhancing effect of NBs within 60 minutes in the following experiments.

**Figure 2:**
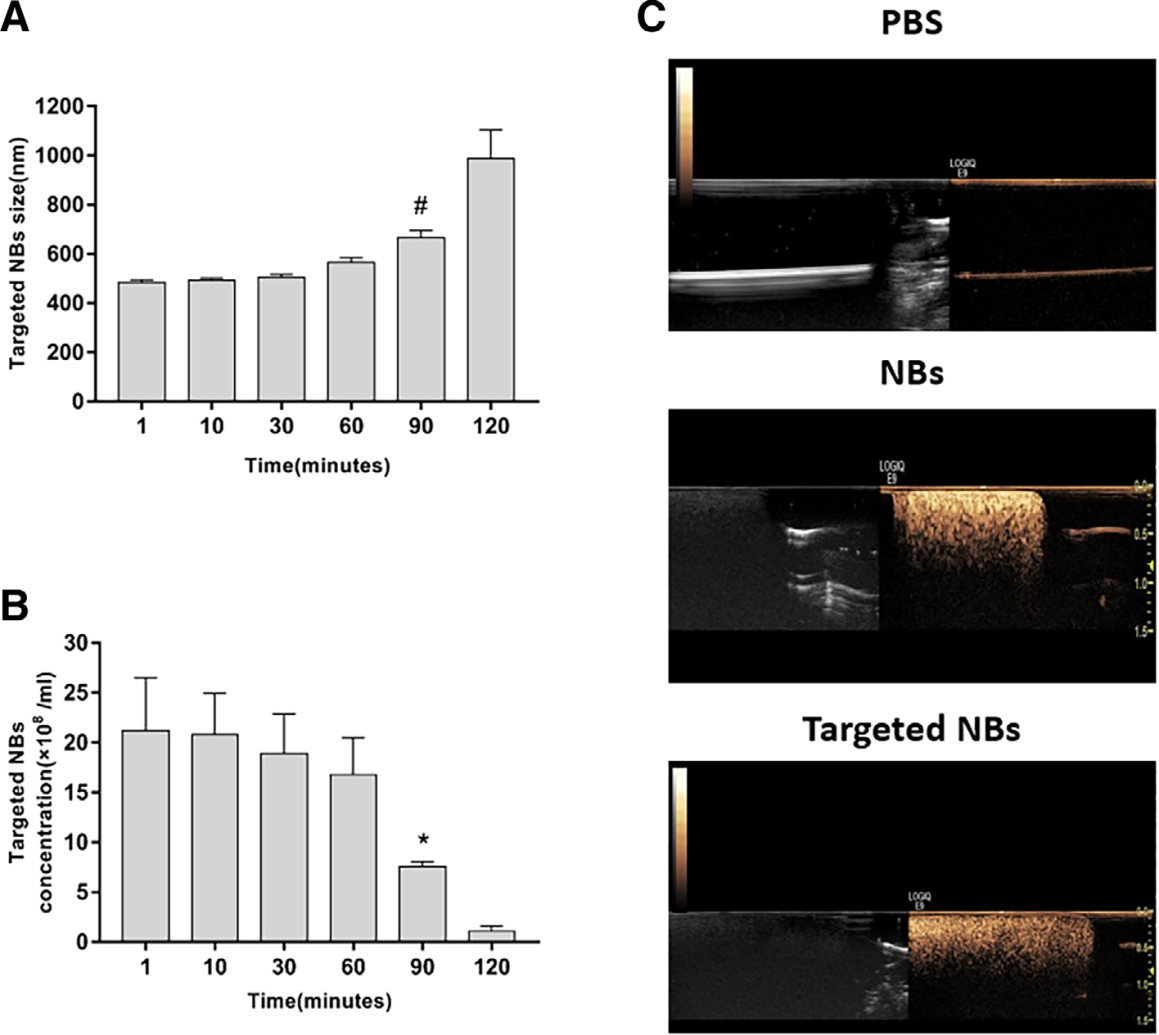
**A** and **B**, Characteristics of the size distribution (**A**) and concentration changes (**B**) of targeted NBs with time. ^#,^*P < .05, significantly different compared with values recorded at 1 minute. **C**, Ultrasound imaging of PBS, NBs, and targeted NBs in vitro.

We subsequently validated the in vitro contrast enhancement that could be achieved using the nontargeted and targeted NBs. Our results showed that, unlike PBS, which was used as a negative control, both the nontargeted NBs and the targeted NBs showed significantly enhanced punctate signals, with a similar capability for superior US contrast enhancement (Figure 2).

### *Cellular Immunofluorescence*

**Figure 3:**
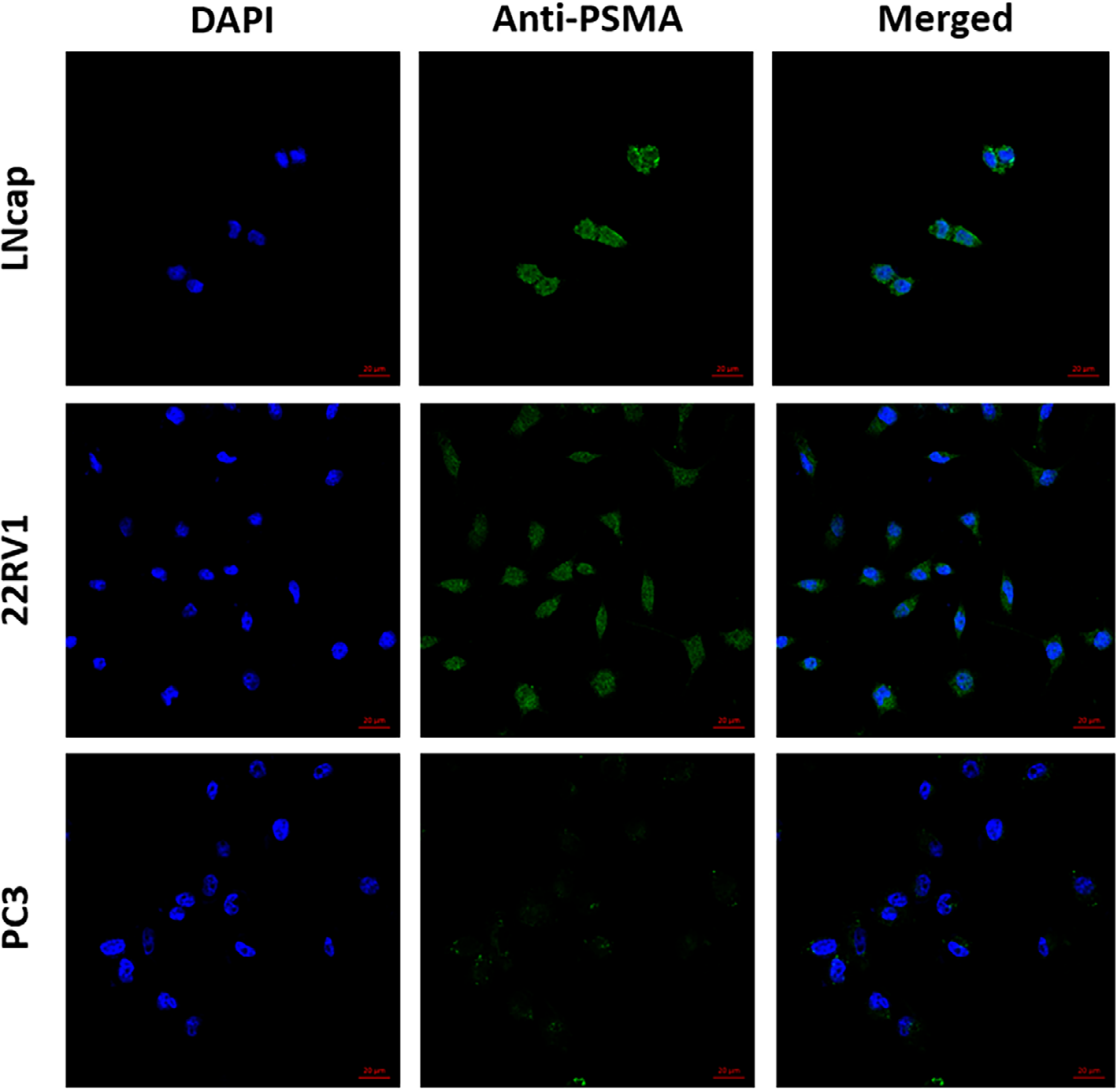
Location of PSMA expression of LNCaP, 22RV1, and PC‐3 prostate cancer cells detected by immunofluorescence assay. DAPI indicates 4′,6‐diamidino‐2‐phenylindole.

The PSMA expression of LNCaP, 22RV1, and PC‐3 cells was verified by immunofluorescence. The nuclei of the 3 cell types showed blue fluorescence under fluorescence microscopy. The LNCaP and 22RV1 cell membranes showed green fluorescence, whereas the PC‐3 cell membranes did not, indicating high PSMA expression in LNCaP and 22RV1 cells and no PSMA expression in PC‐3 cells (Figure 3).

### *In Vitro NB Targeting*

**Figure 4:**
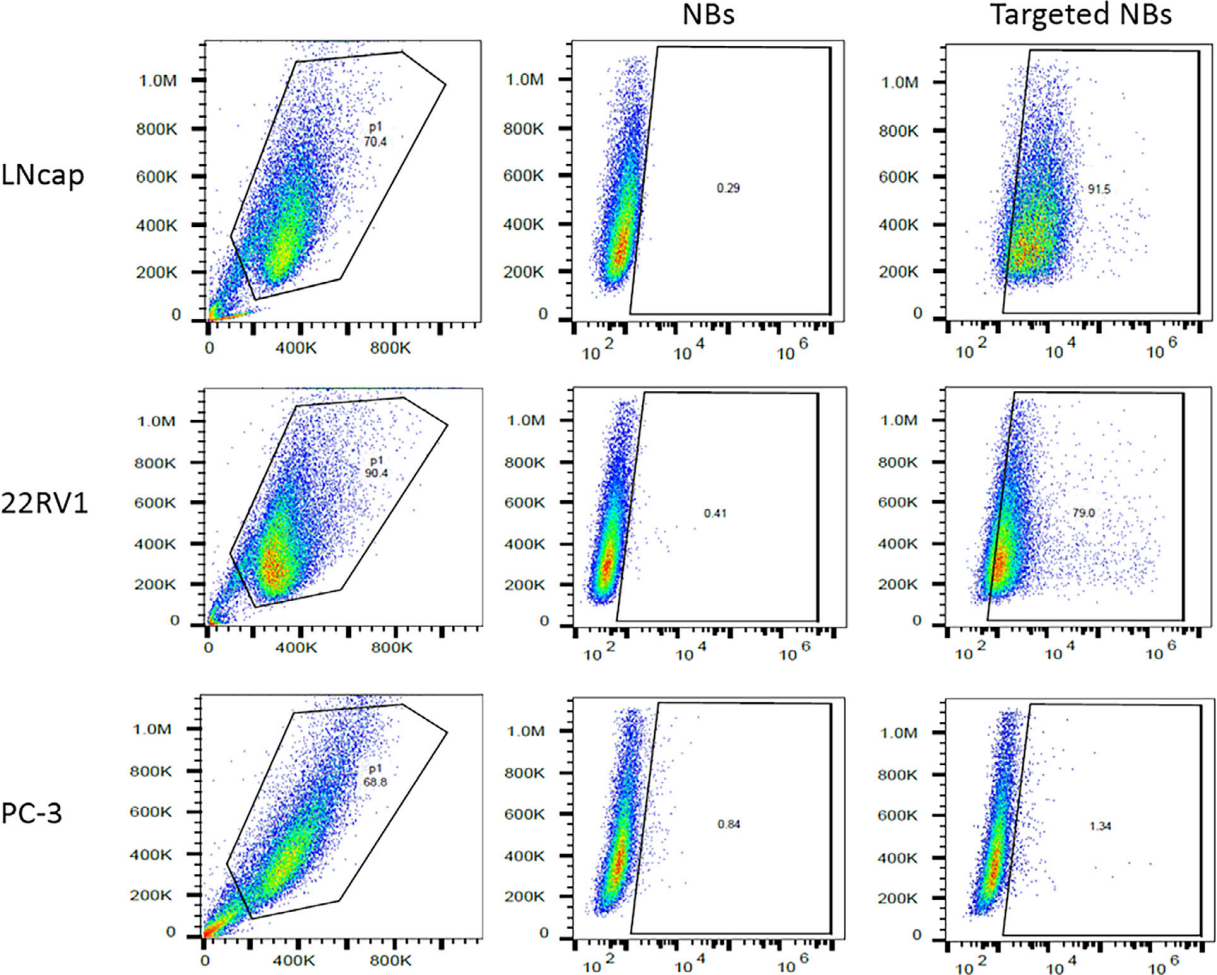
Flow cytometric analysis of the binding rates of LNCaP, 22RV1, and PC‐3 cells with NBs and targeted NBs in vitro.

Flow cytometry was used to compare the ability of the targeted NBs to bind to LNCaP and 22RV1 cells, with high PSMA expression, and PC‐3 cells, with no PSMA expression. A large amount of fluorescence was detected for both the LNCaP and 22RV1 cells in the targeted NB group compared with those in the nontargeted NB control group. Approximately 91.5% and 79% of the LNCaP and 22RV1 cells were bound to the PSMA antibody loaded on the targeted NBs, respectively. However, no fluorescence was observed for the PC‐3 cells, which were negative for PSMA expression (Figure 4).

### *In Vivo US Imaging of Xenograft Tumors*

**Figure 5:**
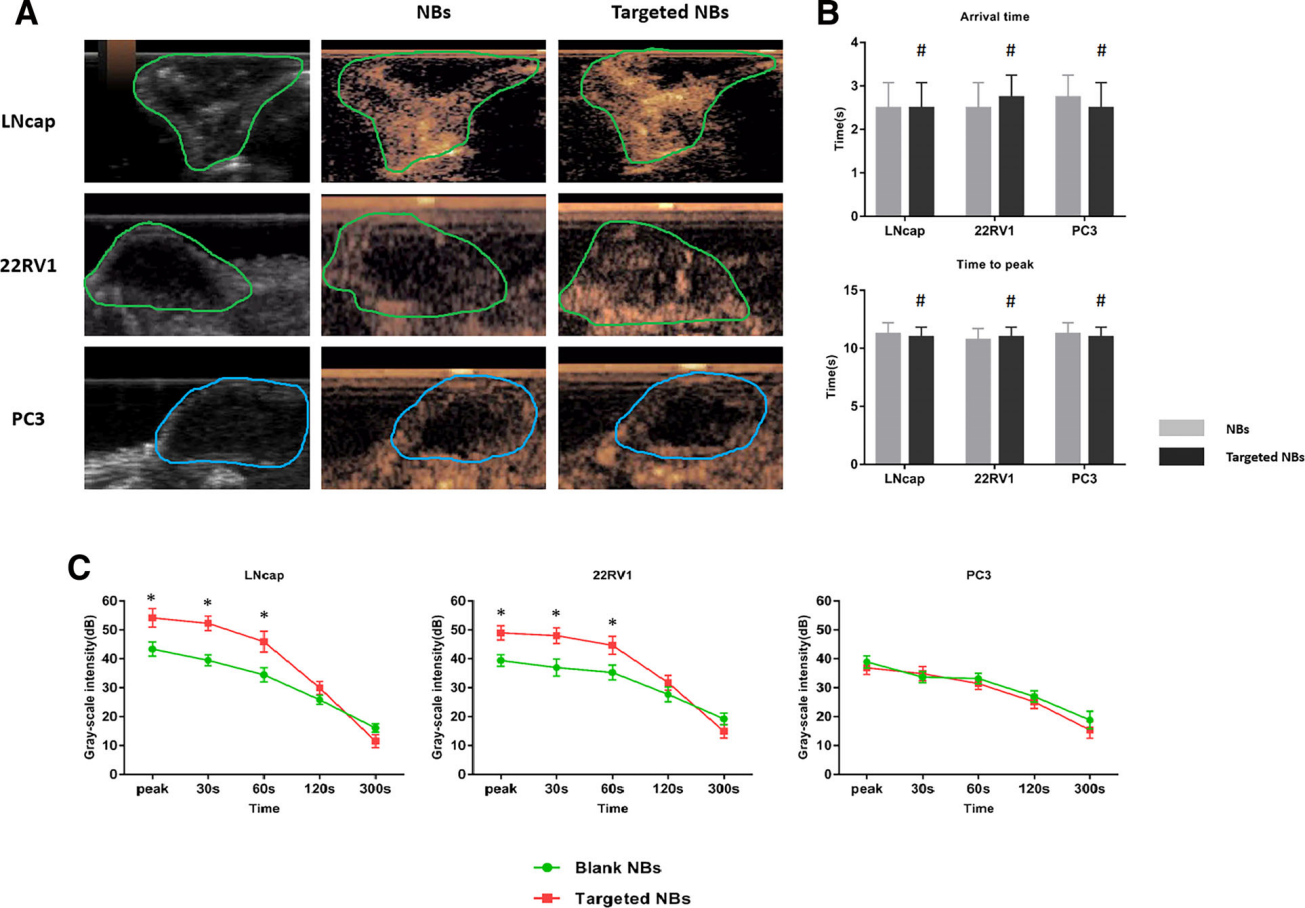
**A**, Contrast‐enhanced images of targeted NBs and blank ones at the time to peak in the 3 transplanted tumors. **B**, Arrival time and time to peak of contrast enhancement of NBs and targeted NBs in the 3 types of transplanted tumors. **C**, Time curve analysis of the contrast‐enhanced image intensity for NBs and targeted NBs in 3 kinds of transplanted tumors. ^#^ *P* > .05, no significant difference between targeted NBs and NBs. *P < .05, significant difference between targeted NBs and NBs.

We examined a total of 20 tumor‐bearing nude mice. The 22RV1 and PC‐3 cell types each tested 8 mice because of the low success rate of tumor bearing in nude mice; the LNCaP cell type tested 4 mice. The imaging characteristics of the nontargeted and targeted NBs were analyzed for each tumor type under the same US conditions. There were no significant differences in the arrival time or the peak time (*P* > .05) between the nontargeted and targeted NBs in the LNCaP, 22RV1, or PC‐3 tumors. Images of the nontargeted and targeted NBs at peak contrast intensity in the tumors were obtained for all 3 tumor types. In the LNCaP and 22RV1 xenografts, the peak contrast intensity of the targeted NBs was significantly higher than that of the nontargeted NBs. However, in the PSMA‐negative PC‐3 xenografts, there was no significant difference in the peak contrast intensity between the NB types. There was a significant difference in the peak contrast intensity between the nontargeted and targeted NBs in the LNCaP and 22RV1 xenografts (*P* < .05), whereas there was no difference in the PC‐3 xenografts (*P* > .05). The results of a further time‐intensity curve analysis are shown in Figure 5. In the LNCaP and 22RV1 xenografts, the targeted NBs had a greater contrast‐enhancing effect within 120 seconds than did the nontargeted NBs. However, at 300 seconds, the enhanced intensity of the targeted NBs was lower than that of the nontargeted NBs; in the PC‐3 xenografts, there was no significant difference in the enhanced intensity between the targeted and nontargeted NBs within 300 seconds (Figure 5). Last, no animals died during the imaging experiment.

### *Confocal Laser‐Scanning Microscopic Examination*

**Figure 6:**
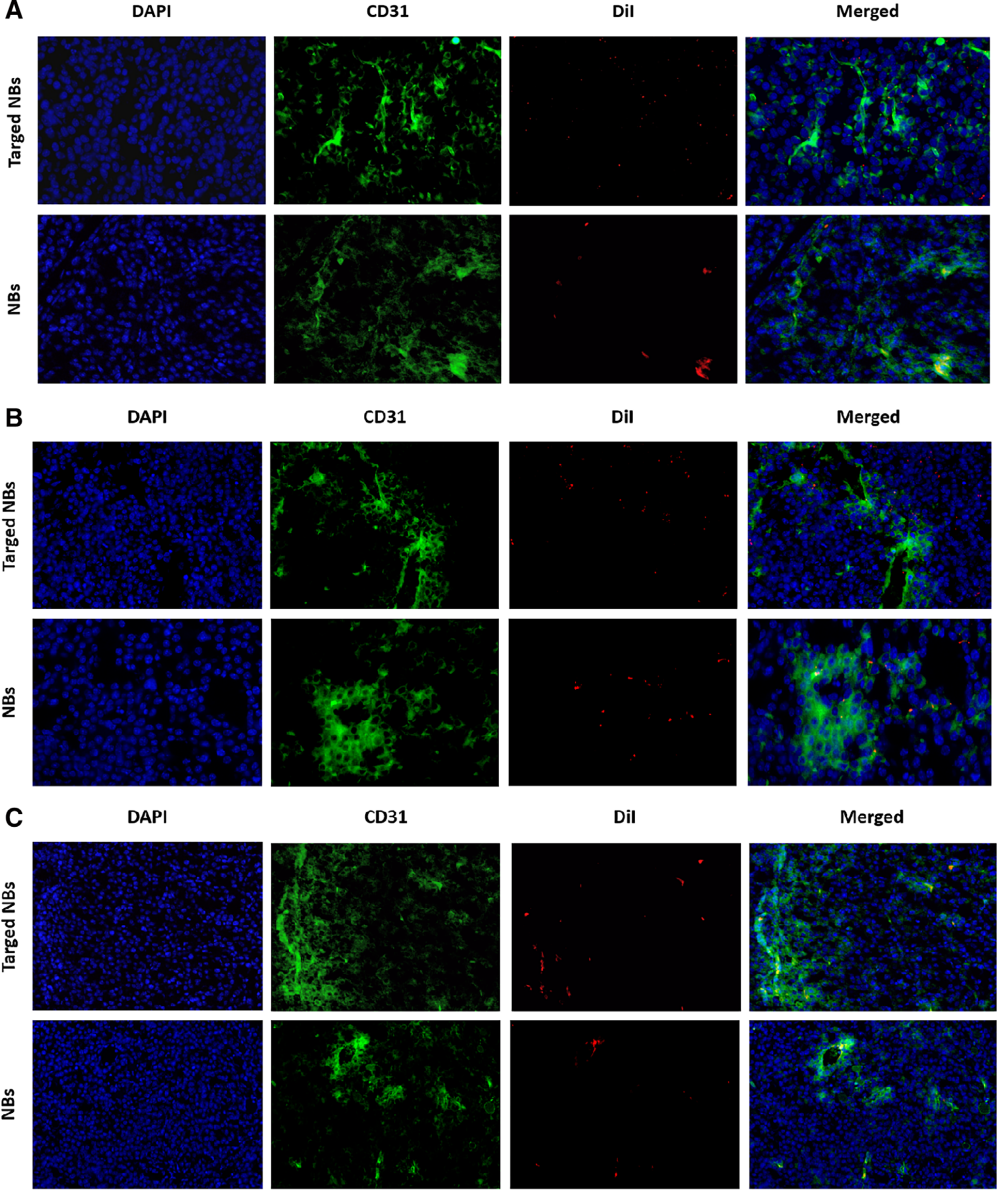
Confocal laser‐scanning microscopic images of frozen sections after nuclei and capillary labeling. **A** and **B**, In LNCaP (**A**) and 22RV1 (**B**) transplanted tumors, NBs and targeted NBs both can be seen in tumor vessels, but targeted NBs on the cell surface are substantially greater than NBs. **C**, In PC‐3 transplanted tumors, NBs and targeted NBs both can be seen in tumor vessels, but few targeted and blank NBs were observed on the surface of PC‐3 tumor cells.

To further compare the microscopic localization of the blank and targeted NBs in tumors, the nude mice were euthanized, and cryosections of the tumors were examined by confocal laser‐scanning microscopy. The results are shown in Figure 6. Both the nontargeted and targeted NBs were observed on the tumor vessels in all 3 types of tumors. Significantly more targeted NBs than nontargeted NBs were observed on the surface of the LNCaP and 22RV1 tumor cells; however, few targeted and blank NBs were observed on the surface of the PC‐3 tumor cells (Figure 6).

## Discussion

Ultrasound has the advantages of low cost, safety, and ease of operation compared to other imaging modalities, such as computed tomography, magnetic resonance imaging, and positron emission tomography.^19^, ^20^ In addition, US can provide real‐time imaging while protecting patients from the risks of radiation.^21^ Therefore, US is often used as one of the preferred methods for diagnosing, treating, and monitoring disease.^5^, ^22^ Prostate transrectal US plays an important role in the diagnosis and treatment of PCa. Transrectal US‐guided biopsy is the reference standard for diagnosing PCa, but there is still the possibility of a missed diagnosis or excessive puncture due to a lack of sensitivity and specificity.^7^, ^23^ Angiogenesis is a fundamental feature of tumor growth and invasion.^24^ Although UCAs, such as MBs, have greatly improved the ability to image the tumor vasculature in recent years, the diagnosis of PCa still lacks specificity. Molecular imaging has become a hot topic in the context of further improving the diagnostic efficacy of US. Proteins, polypeptides, antibodies, polymers, and ligands with specific targeting abilities can be combined with UCAs to indirectly obtain information regarding targeted tissues at the cellular and molecular levels, thus improving diagnostic specificity.^25^, ^26^, ^27^, ^28^

Prostate‐specific membrane antigen is highly expressed in prostatic intraepithelial neoplasia, hormone‐dependent PCa, non–hormone‐dependent PCa, and metastases.^13^, ^14^ The PSMA antibody was developed to specifically bind to PCa cells in vivo,^16^, ^18^ and some studies have shown that the application of PSMA‐targeting or other specific antigen‐targeting MBs can improve the diagnostic accuracy of US.^29^, ^30^, ^31^

Although the vascular endothelial gap is expanded in some disease states, such as in tumors, only particles of less than 700 nm in diameter are allowed to pass through.^32^ Targeted MBs have a wide range of clinical applications, but because they are larger than 1000 μm, it is difficult for MBs to enter tumor tissue through the vessel wall and specifically bind to tumor cells; in addition, the short in vivo half‐life of MBs results in an insufficient time frame for imaging the target.^4^ Therefore, NBs have become a popular topic of recent research on targeted CEUS. Their size of less than 700 nm is advantageous for targeted NBs to reach tumor targets by passing through the vascular endothelium of small, defective tumor blood vessels,^10^, ^11^, ^33^ and in vivo, NBs provide a longer period of enhancement for imaging than MBs.^8^, ^9^ Furthermore, NBs have the advantage of prolonged blood circulation due to their small size and high stability.^34^

The goal of this work was to investigate novel PSMA‐targeting NBs as a targeted UCA for the molecular imaging of PCa. Unlike previous studies of PSMA‐targeting contrast agents,^12^, ^30^ we applied DPPC and biotinylated DSPE‐PEG‐2000 in the lipid shell of the NBs. Microbubbles made of DPPC combine with the ligand to form a dome shape with a larger binding area.^35^ The biotinylated DSPE‐PEG‐2000 has a negative charge,^36^, ^37^ resulting in the prepared NBs being negatively charged and conferring improved stability, dispersibility, and biocompatibility in water. Although PEG is often used for the preparation of lipid shells because it can prolong the serum half‐life of MBs,^38^ studies have shown that PEG may block ligands from reaching target receptors. Interestingly, PEG‐2000 overcomes this disadvantage by having longer chains and fewer random configurations.^39^ At the same time, we found that the thickness of the phospholipid film can be determined by controlling the phospholipid concentration and the size of the flask, and NBs can be directly prepared without the use of differential centrifugation, static stratification, or surfactant addition, as in previous work, to improve the yield and stability of NBs. It is also worth noting that the anti‐PSMA scFv we used was constructed according to a study reported by Friedrich et al.^18^ It has the advantages of strong penetration and high specificity. Meanwhile, unlike most antibodies with a molecular weight of 150 kDa, our anti‐PSMA scFv has a molecular weight of 28 kDa, which ensures that the PSMA scFv‐loaded NBs would still be small enough to pass through the tumor vasculature and reach the tumor cells.

The targeted NBs prepared in this study were less than 700 nm in size, which satisfies the size requirement for passing through the vascular endothelial gap. These NBs also had the advantages of being uniform in size, having good biological activity, and being sufficiently stable for in vivo imaging applications. In vitro FCM experiments showed no significant binding of the nontargeted NBs to any of the 3 cell types, whereas FCM did show significant binding of the targeted NBs to LNCaP and 22RV1 cells, which express PSMA. Approximately 91.5% and 79% of the LNCaP and 22RV1 cells showed green fluorescence, respectively, whereas no significant percentage of the PC‐3 cells showed green fluorescence. On the basis of the above results, we established LNCaP, 22RV1, and PC‐3 xenograft tumors in nude mice and tested the targeted NBs in vivo using CEUS. The targeted NBs showed significantly higher peak contrast intensity values in the LNCaP and 22RV1 xenografts than did the nontargeted NBs, whereas no significant differences were observed between the types of NBs in the PC‐3 xenografts. The results of in vitro experiments at the cellular level and in vivo experiments in xenografts demonstrate that our prepared targeted NBs can more easily pass through tumor capillaries and target tumor cells with high PSMA expression, thereby achieving broader signals than nontargeted NBs on US imaging and producing superior contrast enhancement. In addition, in vivo, the peak contrast intensity of the targeted NBs was higher in the LNCaP xenografts than in the 22RV1 xenografts, which was consistent with the FCM results in vitro, indicating that the contrast enhancement observed with US was positively correlated with the binding of the targeted NBs to tumor cells. Interestingly, the peak contrast intensity of the targeted NBs in the LNCaP and 22RV1 xenografts was lower than that of the nontargeted NBs at 300 seconds, which may have been due to the fact that the targeted NBs were more likely to aggregate in the tumor vasculature and on the surface of tumor cells and thus more likely to rupture. Finally, the immunofluorescence results obtained from tumor sections examined by confocal laser‐scanning microscopy further demonstrated that the targeted NBs could specifically bind to tumor cells after passing through the vascular endothelium. In addition, because NBs may be ruptured or depleted during experimental operations, the number of NBs aggregated in the blood vessels and on the cell surface around the tumor may be much higher than that on a frozen section for tumor pathologic analysis by confocal laser‐scanning electron microscopy.

In conclusion, in this study, we not only prepared uniform lipid NBs but also successfully combined the NBs with a biotinylated anti‐PSMA scFv to obtain targeted NBs with a stable structure and high affinity and specificity for PCa cells with high PSMA expression. We demonstrated in vitro and in vivo that our targeted NBs have the ability to specifically bind to PSMA‐overexpressing PCa cells, and we verified the strong contrast enhancement of the targeted NBs as a UCA. Immunofluorescence examination of tumor cryosections further confirmed the selectivity of the targeted NBs for PSMA‐positive tumors. The targeted NBs prepared in this study have been proven to be an excellent UCA for imaging PSMA‐positive PCa. Thus, these NBs provide a basis for the molecular imaging of PCa by US and have potential as a delivery system for the targeted treatment of PCa.
